# Disseminated Blastomycosis Mimicking Tuberculosis, China

**DOI:** 10.3201/eid3110.250671

**Published:** 2025-10

**Authors:** Can Guo, Yanjing Pan, Jiajia Yu, Linyan Yao, Yuhua He, Junwei Cui, Mengqiu Gao, Yu Pang

**Affiliations:** The First Affiliated Hospital of Xinxiang Medical University, Xinxiang, China (C. Guo, Y. Pan, L. Yao, Y. He, J. Cui); Capital Medical University, Beijing, China (J. Yu, M. Gao, Y. Pang)

**Keywords:** Blastomycosis, Blastomyces percursus, tuberculosis and other mycobacteria, TB, respiratory infections, fungi, diagnosis, China

## Abstract

Blastomycosis is endemic in central and southern North America but rare in China. It can mimic community-acquired pneumonia, tuberculosis, or cancer. We describe a patient who initially had tuberculosis diagnosed and later had blastomycosis diagnosed through metagenomic detection, which aided diagnosis and treatment. Clinicians should consider blastomycosis in differential diagnoses for respiratory diseases.

Blastomycosis is a dimorphic fungal infection endemic to North America but rarely reported in China. (*1*). The pathogen is not directly transmissible between persons (*1*). Disseminated blastomycosis should be considered in cases with multisystemic involvement, even when typical symptoms such as cutaneous or central nervous system lesions are absent (*2*). Clinical manifestations of blastomycosis often mimic those of tuberculosis (TB), malignancy, or bacterial pneumonia, complicating diagnosis. *Blastomyces percursus*, a novel species phenotypically and epidemiologically distinct from *B. dermatitidis*, has been linked to infections in patients from Israel, South Africa, and other regions in Africa (*3*,*4*). We report an unusual case of *B. percursus* infection in China that was initially diagnosed as pulmonary tuberculosis.

A 38-year-old man from Xinxiang, Henan Province, China, sought care after 4 months of cough, hemoptysis, and weight loss. He had been treated empirically with antimicrobial drugs at a local hospital without substantial improvement. He had no history of incarceration, foreign travel, or known immunosuppression. He worked as a truck driver and had lived locally for years.

Chest computed tomography scans revealed bilateral lobar infiltrates and patchy opacities (Figure 1, panels A, B). Tests for *Aspergillus* *galactomannan*, β-D-glucan, and respiratory viruses were negative. Bronchoscopy revealed noncaseating granulomatous inflammation, and all stains and cultures for mycobacteria and fungi were negative. Because of clinical suspicion, we started the patient on a 4-drug TB treatment regimen (rifampin 600 mg, isoniazid 300 mg, ethambutol 1,000 mg, and pyrazinamide 1,500 mg daily).

**Figure 1:**
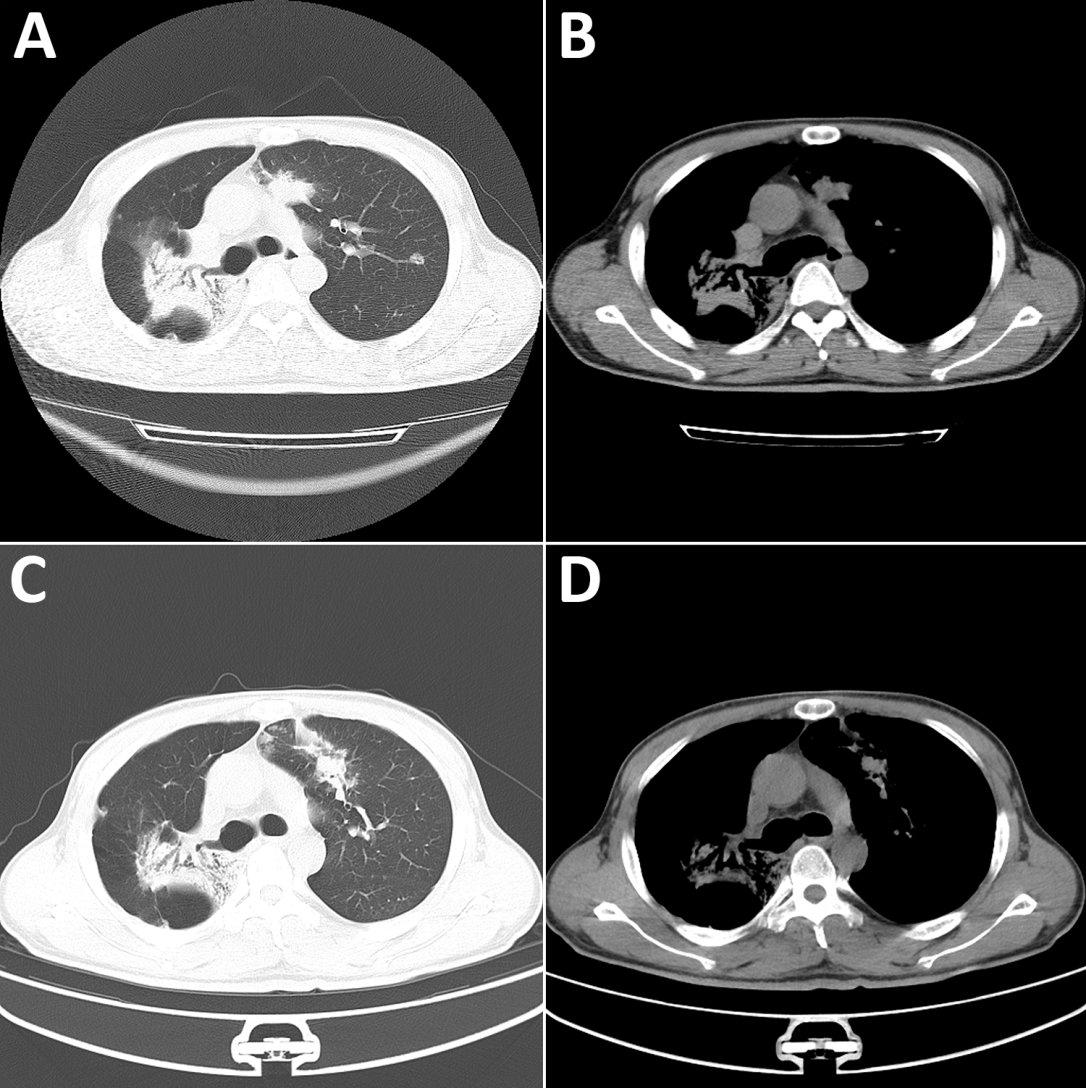
Chest computed tomography (CT) images from a patient with disseminated blastomycosis mimicking tuberculosis, China. A, B) Chest CT at first admission revealed bilateral lobar infiltrates (A) and patchy opacities (B). C, D) Chest CT taken 2 months later showed resolution of left lung lesions but progression of right lung lesions.

Two months later, the patient was readmitted with new cutaneous lesions on his abdomen and lower limbs, which were nodular and verrucous (Figure 2). Laboratory studies showed leukocytosis (leukocyte count 14,160/μL) and 85.9% neutrophils. Results of ELISA testing for HIV were negative, and results for tumor markers and autoimmune antibody panels were unremarkable. Repeat chest computed tomography scans demonstrated resolution of left lung lesions but progression on the right (Figure 1, panels C, D).

**Figure 2:**
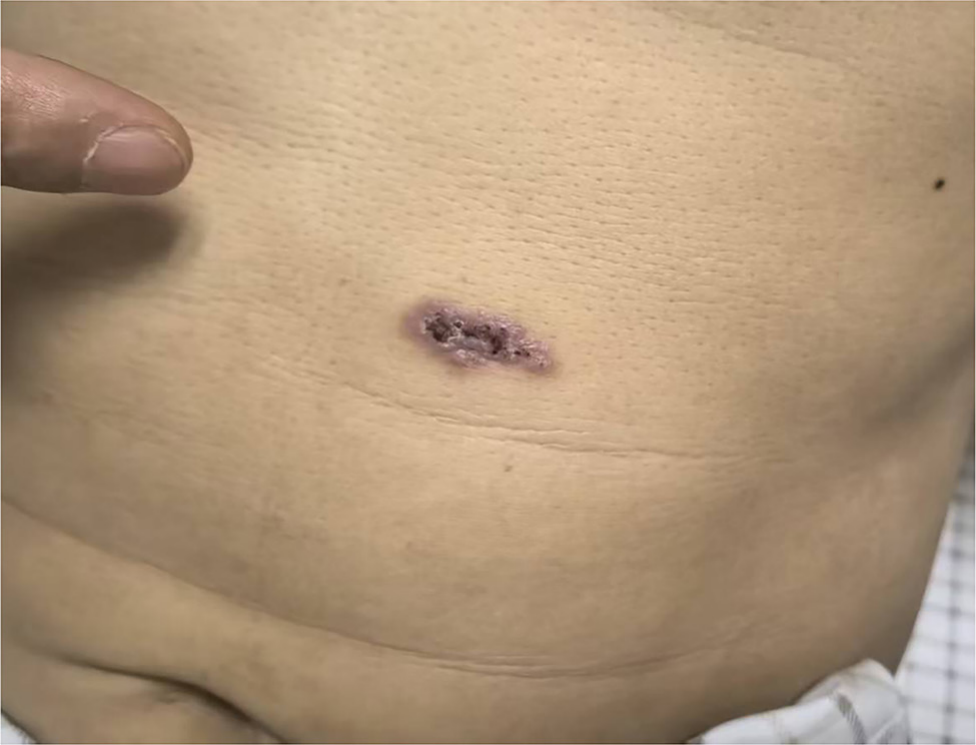
Cutaneous lesion on the abdomen from a case of disseminated blastomycosis mimicking tuberculosis, China. Two months after treatment for tuberculosis, new cutaneous lesions developed on the patient’s abdomen and lower limbs; molecular methods on bronchoalveolar lavage fluid detected *Blastomyces percursus*.

Repeat bronchoscopy was performed. GeneXpert MTB/RIF (Cephalid, https://www.cepheid.com) testing of bronchoalveolar lavage fluid was negative. However, 1 week later, metagenomic next-generation sequencing of bronchoalveolar lavage fluid identified *B. percursus*.

The patient received intravenous liposomal amphotericin B (3 mg/kg/d) for 2 weeks, then oral itraconazole (200 mg 2×/d) for 1 month. After treatment, both pulmonary and cutaneous lesions resolved. Itraconazole remains a preferred agent for treating mild-to-moderate pulmonary and disseminated blastomycosis (*8*).

Blastomycosis can involve virtually any organ and often masquerades as TB, leading to frequent misdiagnoses. In this case, the patient was treated with multiple drugs for TB, delaying accurate diagnosis by >1 month. Ultimately, metagenomic sequencing identified *B. percursus*. Metagenomic approaches increasingly have been applied to clinical microbiology for detecting pathogens in respiratory infections (*5*), bloodstream infections (*6*), and central nervous system infections (*7*). Those methods make unbiased, broad-spectrum detection possible even when conventional tests fail.

The delay in diagnosis led to unnecessary drug exposure and progressive dissemination, including cutaneous lesions. Medications for TB carry substantial risks, including hepatotoxicity and bone marrow suppression. The lack of reliable rapid diagnostic tests for *B. percursus* remains a barrier to timely treatment. Comprehensive physical examination, especially of the skin, is crucial, because cutaneous findings can aid in early recognition. In this case, clinicians adhered to a TB diagnosis despite a lack of microbiological evidence, and skin lesions developed in the patient while he was receiving empirical TB therapy only. Earlier consideration of alternative etiologies, such as blastomycosis, nocardiosis, or actinomycosis, might have expedited appropriate care.

This case illustrates how blastomycosis, although rare in China, can mimic pulmonary TB and lead to delayed diagnosis and treatment. Clinicians should include blastomycosis in the differential diagnosis of unexplained pulmonary infections. Increasing awareness of endemic mycoses and integrating metagenomic tools into routine diagnostics could improve the precision and timeliness of care.
